# Supplementary material for: Absence of Aquaporin-4 (AQP4) Prolongs the Presence of a CD11c+ Microglial Population during Postnatal Corpus Callosum Development
Source: Int J Mol Sci. 2024 Jul 30;25(15):8332. doi: 10.3390/ijms25158332 (PMC11312288; doi:10.3390/ijms25158332)
Supplement: Supplementary file 1 [file ijms-25-08332-s001.zip › ijms-3106949-supplementary.pdf]

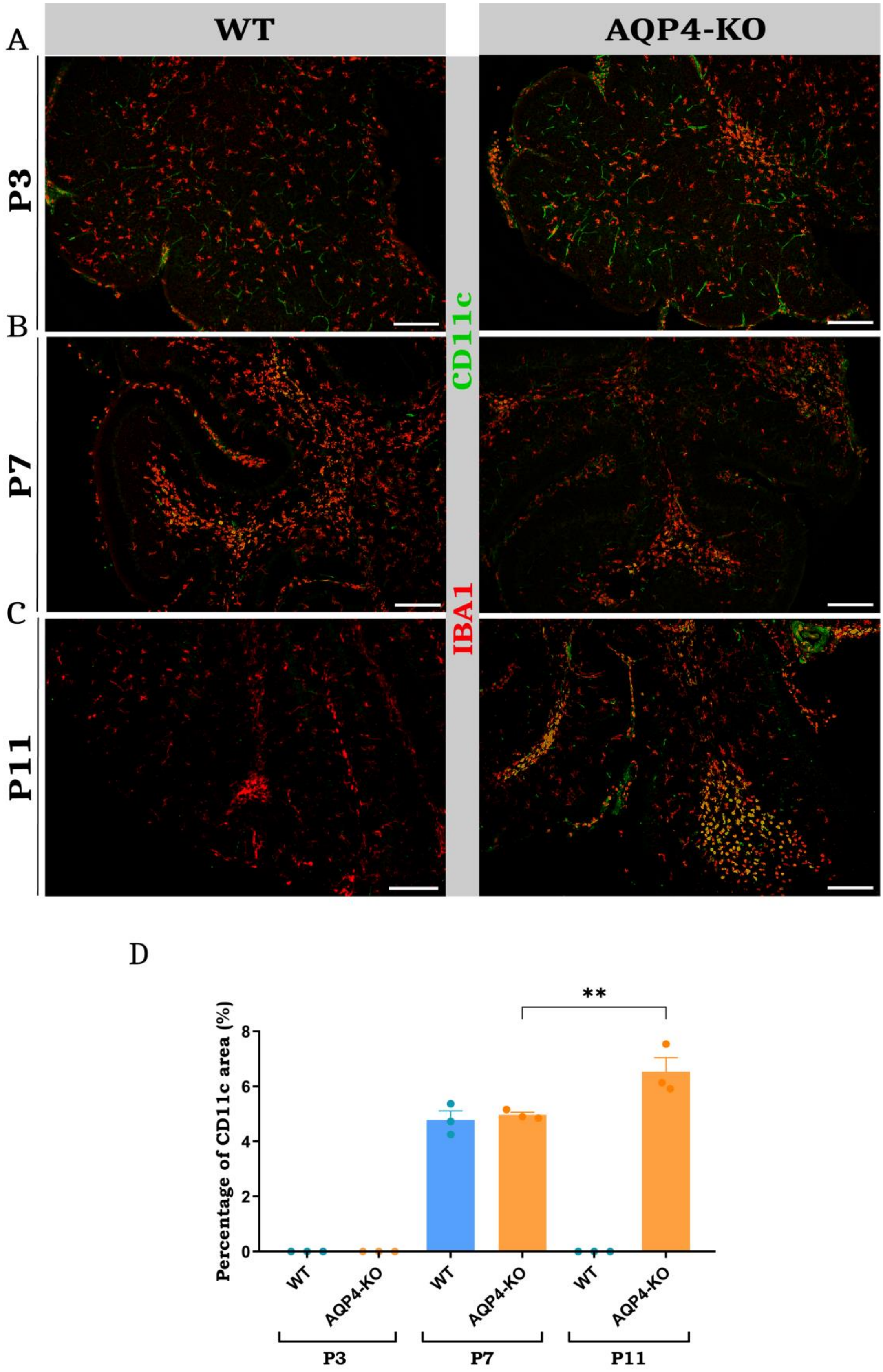

Supplementary. Figure S1. Temporal expression pattern of the CD11c+ microglial subtype in the cerebellum during the early postnatal period. Compositions of fluorescence microscopy images to determine the presence of CD11c+ microglial cells comparatively (WT vs AQP4-KO) at developmental stages P3 (A), P7 (B) and P11 (C). Scale bars = 200 µm. (D) Quantifications of microglial cell abundance in the evaluated sections. N=3 samples per condition. Significant differences between groups were assessed using one-way ANOVA followed by Tukey's post hoc test (*p<0.05).
